# Sociodemographic and behavioural risk factors associated with low awareness of diabetes mellitus medication in Indonesia: Findings from the Indonesian Family Life Survey (IFLS-5)

**DOI:** 10.3389/fpubh.2023.1072085

**Published:** 2023-01-25

**Authors:** Qisty A. Khoiry, Sofa D. Alfian, Rizky Abdulah

**Affiliations:** ^1^Department of Pharmacology and Clinical Pharmacy, Faculty of Pharmacy, Universitas Padjadjaran, Sumedang, Indonesia; ^2^Center of Excellence for Pharmaceutical Care Innovation, Universitas Padjadjaran, Sumedang, Indonesia

**Keywords:** awareness medication, diabetes mellitus, determinants, IFLS, Indonesia

## Abstract

**Introduction:**

Low awareness of the necessity of taking medication is common among patients with diabetes mellitus (DM) due to their lack of understanding of the disease. Therefore, it is essential to determine the underlying risks influencing low awareness to design effective intervention strategies. This study aims to evaluate the association of sociodemographic and behavioural factors with low awareness to take medication among patients with DM in Indonesia.

**Method:**

Retrospective data were obtained from the Indonesian Family Life Survey (IFLS-5), a national cross-sectional population-based survey among respondents with DM aged ≥15 years. DM status was confirmed by HbA1c testing, while sociodemographic and other health-related information was obtained from self-reported data. Gender, age, educational level, marital status, economic status, comorbidity, religiosity, residence and health insurance status were considered sociodemographic, whereas blood glucose monitoring status, sleeping problems, depression status, having a general medical check-up, satisfaction with healthcare needs and happiness status were considered behavioural risk factors. Awareness of DM medication was determined by self-reported responses to the question asked by the surveyor. Logistic regression analysis was used to evaluate the association between sociodemographic and behavioural factors and low awareness of DM medication. Odds ratios (ORs) with 95% confidence intervals (CIs) were reported.

**Result:**

Most of the 706 respondents were female (58.8%) and aged 55–65 years (28.8%). Most of them showed low awareness of diabetes medication (87.7%). Irregular blood glucose monitoring (OR: 23.61, 95% CI 11.46–48.65; *p* < 0.001), without any comorbidity (OR: 2.03, 95% CI 1.05–3.90; *p* = 0.034), never had any general medical check-up (OR: 2.52, 95% CI 1.12–5.36; *p* = 0.016), 26–35 years of age (OR: 4.96, 95% CI 1.06–23.19; *p* = 0.042), 36–45 years of age (OR: 5.04, 95% CI 1.17–21.69; *p* = 0.030) and having no health insurance coverage (OR: 2.08, 95% CI 1.12–3.87; *p* = 0.021) were significantly associated with low awareness of diabetes medication.

**Conclusion:**

Healthcare professionals should regularly evaluate blood glucose level, perform routine medical check-ups, prioritise patient satisfaction by providing appropriate care, involve patients in decision-making by determining their needs and then tailor an intervention to meet the need for, and improve their awareness of, DM medication.

## 1. Introduction

Diabetes mellitus (DM) has increased drastically in the past three decades; around 422 million people worldwide have diabetes, with deaths totalling 1.5 million (1). The number of patients with DM in Southeast Asia is predicted to increase by 113 million by 2030 (2). DM ranks second among the most common non-communicable diseases in Indonesia, with a prevalence of 10.9% (3). In addition, more than half of the individuals with DM in Indonesia (73.7%) were unaware of their condition (4). Therefore, if DM is left unmanaged and untreated, it could lead to either microvascular (retinopathy, nephropathy and neuropathy) or macrovascular (stroke, cardiovascular disease and peripheral artery disease) complications (5). Unfortunately, no complete cure for diabetes has been found; thus, long-term treatment to prevent or delay complications and maintain the patient's quality of life is needed (6).

Healthcare professionals have a crucial role in developing strategies to facilitate medication adherence so that patients can optimise diabetes treatment and limit the progression of diabetes (6). This emphasises the significance of patient education and awareness of DM medication after having acquired awareness of DM (7). Thus, the first approach for healthcare practitioners could be to increase DM medication awareness. Awareness of medication was defined as a patient's common knowledge or understanding about his/her medication without direct instruction or as a sort of medication self-consciousness (8). Awareness is one of the five primary types of related concerns with the potential to improve therapy since they are obstacles to medication adherence from the patient's perspective (9). It is somewhat distinct from medication adherence, defined as the process whereby patients take their medication as prescribed (10). Inadequate medication adherence among DM patients remains a major problem leading to disease progression (11). At least 45% of treatment failures among DM patients are caused by low adherence to anti-diabetic medical treatments (12). This could lead to increases in health expenses yearly at both patient and societal levels (13).

Patients with good comprehension and high awareness of diabetic medication exhibited an improvement in their glucose control (14) and medication adherence (15). Therefore, identifying factors associated with medication awareness is an important first step to improving medication adherence. Although studies about medication awareness in patients with diabetes are limited, some studies have explored a positive correlation between the frequency of healthcare professional counselling and the patient's level of awareness to take their medication (16, 17). The Indonesian Family Life Survey (IFLS) is a longitudinal socioeconomic and health survey in Indonesia, which covers ~83% of the Indonesian population (18). Previous studies that employed IFLS-4 or IFLS-5 mostly examined the prevalence of DM and its sociodemographic risk factors (19–22). Other research evaluated the association between socioeconomic characteristics and the incidence of diabetes (23, 24). Until now, no study has investigated psychosocial and sociodemographic factors associated with medication awareness among DM patients using IFLS. It is still unclear as to which components or focal points are necessary to increase DM medication awareness in Indonesia. Therefore, addressing its fundamental causes is vital for developing effective intervention strategies. This study aims to identify sociodemographic and behavioural factors associated with low awareness of DM medication in Indonesia.

## 2. Methods

### 2.1. Study design

The cross-sectional study design utilised in this study is based on secondary data, IFLS-5. The IFLS is a longitudinal study that used a multistage stratified sample design to represent 83% of the Indonesian population (18). The IFLS sampling strategy stratified on provinces and urban/rural locations and then sampled randomly within these strata. Provinces were chosen to optimise population representation, represent Indonesia's cultural and socioeconomic variety and be cost-effective to survey, given the country's size and geography. Therefore, 13 of the 27 provinces that existed at the time were included. The IFLS randomly selected 321 enumeration areas (EA) within each of the 13 provinces, oversampling urban EAs and EAs in smaller provinces to facilitate urban–rural and Javanese–non-Javanese comparisons. Twenty households from each urban EA and 30 households from each rural EA were selected (18). The IFLS gathered sociodemographic, economic, and health status characteristics, including self-reported health status, symptoms and pain assessments, as well as biomarker assessments. Preliminary testing of the IFLS questionnaire ensured its reliability and validity before the full-scale survey was conducted (18). The ethical review boards of the RAND's Human Subjects Protection Committee (s0064-06-01-CR01) approved IFLS research. Before data collection, every respondent provided written consent (18). Approval was sought from the research ethics committee of Universitas Padjadjaran, Indonesia, which waived the requirement because this study uses anonymous data from the IFLS.

### 2.2. Study population

Data were collected from IFLS-5 from individuals aged at least 15 years after the survey. Individuals with available data on HbA1c and medication for chronic diseases were included.

### 2.3. Outcome measure

Patients with DM were defined as having an HbA1c value of ≥6.5% (25). The blood samples examined were obtained from dried blood spots (DBSs) and taken through the capillaries at the fingertips (18). These blood samples are easier to obtain than those taken through intravenous vessels and are more durable in terms of storage (26). However, the results of the HbA1c examination from DBS will first be converted to whole blood HbA1c, the gold standard of HbA1c examination, so that it can be used in the diagnosis of DM (18).

Awareness of DM medication was determined by the responses to the following question posed by the surveyor: *Are you currently taking prescription medication weekly to manage your DM?* Those who responded with a “yes” were considered to have a good awareness of DM medication, whereas those who responded with a “no” were thought to have low awareness. This questionnaire has been validated and involved extensive pretests and analysis of the pretest data (18).

### 2.4. Potential factors associated with low awareness of DM medication

Sociodemographic and behavioural risk factors were analysed as potential contributors to low awareness of DM medication. Age, gender, educational level, marital status, residency, economic status, health insurance coverage, religiosity and comorbidity status were categorised into sociodemographic and behavioural factors. At the same time, behavioural factors included blood glucose monitoring status, general medical check-ups, health care satisfaction, happiness status, insomnia and depressive symptom status.

Sociodemographic factors were age after the survey, gender (male/female), level of education (no education, elementary school, junior high school, senior high school and university), marital status (currently married and currently unmarried), residency (urban and rural) and health insurance coverage (yes and no). To assess the economic status, we divided the annual household income in rupiah by family size during the previous 12 months (per capita income). Capita income was categorised per quintile. The quintile is categorised by sorting the per capita income from lowest to highest and then dividing them into five equal groups (the first quintile = an income ≤ $77.01; the second quintile = $77.01–$256.70; the third quintile = $256.70–$483.55; the fourth quintile = $483.55–$924.7; the fifth quintile ≥ $924.7). To evaluate religiosity, we asked the question *How religious are you?* The individuals who said they were extremely religious or religious were classified as religious, whereas those who said they were somewhat religious or not religious were classified as non-religious (27).

Depressive symptoms were assessed using a self-reported Centre for Epidemiologic Studies Depression (CES-D) scale (18). The CES-D consists of 10 items highly linked to the presence of depressive symptoms (28). Eight of the questions examine the negative symptoms of depression (e.g., *I felt fearful and lonely*), whereas the other two examined the positive symptoms (e.g., *I felt hopeful about the future)*. The respondents stated how frequently each item applied to them in the previous week using a four-point Likert-type scale (0 = rarely or never, 1 = some or little, 2 = moderately or much of the time, and 3 = frequently or almost always). After reversing the positive mood items, the total score is obtained by summing all elements. An individual with a total score of ≥10 is deemed to have depressive symptoms (29). The CES-D questionnaire was translated into Indonesian (forward translation) and then re-translated separately into English by two translators (back translation) (18).

Ten Patient-Recorded Outcomes Measurement Information System (PROMIS) questions were used to assess the severity of insomnia (18). Each measure of sleep quality and sleep impairment during the previous week was determined using a set of five items (30). Each item was rated using a five-point Likert-type scale (0 = never/not at all; 1 = a little bit; 2 = somewhat; 3 = quite a bit; 4= always/very much). Insomnia was defined as a total score of ≥21–40 (31). The PROMIS questionnaire was translated into Indonesian (forward translation) and then re-translated separately into English by two translators (back translation) (18).

The question *Has a doctor/paramedic/nurse/midwife ever told you that you have the following chronic ailments or diseases?* was used to evaluate whether the individuals had comorbid disease status, with the following potential responses: hypertension; DM; TB; asthma and other chronic lung diseases; cardiac disease (heart attack/coronary heart disease/angina or other heart diseases); liver disease; stroke; cancer or other malignancies; gout/uric acid; depression and vision and hearing abnormalities (27). Individuals who responded only with DM were classified as having no comorbidity. Individuals who responded with DM and one to three additional chronic diseases were classified as having 1–3 comorbidities, whereas individuals with four or more comorbidities were classified as having >4 comorbidities.

Individuals' response to the question *How regularly do you have your blood glucose checked?* determined the classification of blood glucose monitoring status as either regular or irregular. Individuals' response to *Have you had a general check-up in the recent 5 years?* as either yes or no determined one's status as having/not having had a general medical check-up. Happiness level was determined by asking *Overall, how would you describe the current state of these days? Would you say you are very happy, happy, unhappy or very unhappy?* (27). Those who responded with very happy or happy were categorised as happy, whereas those who responded with unhappy or very unhappy were categorised as unhappy. Satisfaction with healthcare was measured with the question *In relation to your healthcare, which of the following is true: it is less than adequate for my needs; it is just adequate for my needs or it is more than adequate for my needs?* (27). Low healthcare satisfaction corresponds to the response “it is just less than adequate for my needs,” and high healthcare satisfaction corresponds to “it is just adequate for my needs” or “it is more than adequate for my needs.”

### 2.5. Statistical analysis

Descriptive statistics were used to summarise the characteristics of the individuals. Awareness of DM medication was estimated for each age group and gender. The Little test was performed to assess whether the incomplete data type was classified as Missing Completely At Random (MCAR). The Little test assumes that the missing-ness of the data is independent of both observed and unobserved data, thus, a *p* > 0.05 is considered MCAR because it is asymptotically distributed under the null hypothesis that there are no differences between the observed and unobserved data (32). Since the missing data were MCAR (*p* > 0.005), complete case analyses could be carried out (32). A Chi-square test was conducted to evaluate the bivariate relationship between the individuals' characteristics and outcomes. The potential factors related to the outcome in the bivariate analysis at a significance threshold of *p* < 0.25 were included in the initial multivariate model. To determine the odds ratio (OR) with a 95% confidence interval (95% CI), multivariate logistic regression with manual backward elimination was used. The p-values for the factors included in the final model were all fixed to *p* < 0.05. The Hosmer–Lemeshow test was used to assess the goodness-of-fit statistic; R-squared is a number ranging from 0 to 1, indicating how much the combination of independent factors influences the value of the dependent variable at the same time (33). All statistical analyses were performed using Stata software version 14.0 for Windows.

## 3. Results

### 3.1. General characteristics of the study population

A total sample of 706 individuals without missing data on awareness of DM medication was included in this study. The majority of the respondents were female (58.8%) and aged 55–65 years (28.8%; Table 1). A high proportion of individuals had irregular blood glucose monitoring (90.1%), with most of them (88.4%) not having undergone a general medical check-up. Approximately half of the individuals were not covered by health insurance (50.4%) and had no comorbidities (50.1%). Out of the total respondents, 22.1% experienced depression, whereas 85.9% had insomnia.

**Table 1:** Baseline characteristics of the study population.

**Characteristic**	**Low awareness of diabetes mellitus medication**		**High awareness of diabetes mellitus medication**		***p*-value**	**Total respondent (*****n*** = **706)**	
	***n***	**%**	***n***	**%**		***n***	**%**
Total respondent	619	87.7	87	12.3		706	100%
Gender					0.467		
Female	348	87.9	48	12.1		396	58.8
Male	239	86.0	39	14.0		278	41.2
Missing	32	–	–	–		32	–
Age					< 0.001^*^		
15–25 years old	48	100	0	0		48	7.1
26–35 years old	85	96.6	3	3.4		88	13.0
36–45 years old	103	97.2	3	2.8		106	15.7
46–55 years old	89	82.4	19	17.6		108	16.0
55–65 years old	153	78.9	41	21.1		194	28.8
>65 years old	109	83.8	21	16.2		130	19.3
Missing	32	–	–	–		32	–
Education level					0.237^*^		
No education	73	93.6	5	6.4		78	11.8
Elementary school	227	88.0	31	12.0		258	38.9
Junior high school	93	86.1	15	13.9		108	16.3
Senior high school	10	83.3	2	16.7		12	1.8
University	173	83.6	34	16.4		207	31.2
Missing	43	–	–	–		43	–
Marital status					0.105^*^		
Not currently Married	163	90.6	17	9.4		180	26.7
Currently married	424	85.8	70	14.2		494	73.3
Missing	32	–	–	–		32	–
Economic status					0.231^*^		
Quintile 1	127	87.6	18	12.4		153	22.7
Quintile 2	103	86.5	16	13.5		107	15.9
Quintile 3	120	88.2	16	11.8		134	19.9
Quintile 4	111	91.7	10	8.3		126	18.7
Quintile 5	126	82.3	27	17.7		154	22.8
Missing	32	–	–	–		32	–
Residency					< 0.001^*^		
Rural	247	93.6	17	6.4		264	37.4
Urban	372	84.2	70	15.8		442	62.6
Missing	–	–	–	–		–	–
Coverage of health insurance					< 0.001^*^		
No	320	91.2	31	8.8		351	52.6
Yes	261	82.6	55	17.4		316	47.4
Missing	38	–	1	–		39	–
Religiosity					0.916		
Not religious	86	86.9	13	13.1		99	16.3
Religious	445	87.2	65	12.8		510	83.7
Missing	88	–	9	–		97	–
Having comorbidities	< 0.001^*^		
No comorbid	313	93.7	21	6.3		334	50.1
1–3 Comorbidities	263	81.1	61	18.9		323	48.4
>4 Comorbidities	5	55.6	4	44.4		10	1.5
Missing	38	–	1	–		39	–
Blood glucose control status					< 0.001^*^		
Irregularly	514	93.3	37	6.7		551	90.3
Regularly	18	30.5	41	69.5		59	9.7
Missing	87	–	9	–		96	–
Having general medical check-up					< 0.001^*^		
No	484	89.8	55	10.2		539	88.4
Yes	48	67.6	23	32.4		71	11.6
Missing	96	–	–	–		96	–
Health care satisfaction	0.836		
Low	122	87.8	17	12.2		139	22.7
High	412	87.1	61	12.9		473	77.3
Missing	85	–	9	–		94	–
Happiness status					0.726		
Unhappy	62	88.6	8	11.4		70	11.4
Happy	472	87.1	70	12.9		542	88.6
Missing	85	–	9	–		94	–
Insomnia					0.994		
Yes	75	87.2	11	12.8		525	85.9
No	458	87.2	67	12.8		86	14.1
Missing	86	–	9	–		95	–
Depressive symptoms status					0.213^*^		
Not depressed	410	86.3	65	13.7		475	77.9
Depressed	122	90.4	13	9.6		135	22.1
Missing	87	–	9	–		96	–

^*^Included in the initial multivariate model.

### 3.2. Risk factor of low awareness of DM medication

The prevalence of low awareness of DM medication was 87.9% in females and 86.0% in males. Gender, educational level, marital status, economic status, religiosity, happiness status, insomnia and depressive symptoms were not statistically significant differences between the low-awareness DM medication group and the high-awareness DM medication group (Table 1). Age, residency, health insurance coverage, blood glucose monitoring status, comorbidities and general medical check-up were selected as potential factors associated with low awareness of DM medication on the basis of bivariate analyses. In the multivariate model, irregular blood glucose monitoring (OR: 23.61, 95% CI 11.46–48.65; *p* < 0.001), having no comorbidity (OR 2.03, 95% CI 1.05–3.90; *p* = 0.034), not having undergone any general medical check-up (OR 2.52, 95% CI 1.12–5.36; *p* = 0.016), 26–35 years of age (OR 4.96, 95% CI 1.06–23.19; *p* = 0.042), 36–45 years of age (OR 5.04, 95% CI 1.17–21.69; *p* = 0.030) and having no health insurance coverage (OR 2.08, 95% CI 1.12–3.87; *p* = 0.021) were significantly associated with low awareness of diabetes medication (Table 2). The goodness-of-fit *p*-value of the model was 0.552, with an R-squared value of 34.71%.

**Table 2:** Association between sociodemographic and behavioural factors and low awareness of diabetes mellitus medication.

**Characteristic**	**Univariate**		**Multivariate** ^**a**^	
	**Crude OR** **[95% CI]**	***p*-value**	**Adjusted OR [95% CI]^b^**	***p*-value**
**Age**				
15–25 years old	1		1	
26–35 years old	5.46 [1.58–18.91]	0.007^*^	4.96 [1.06–23.19]	0.042^*^
36–45 years old	6.61 [1.92–22.84]	0.003^*^	5.04 [1.17–21.69]	0.030^*^
46–55 years old	0.90 [0.46–1.78]	0.768	1.07 [0.41–2.76]	0.895
55–65 years old	0.72 [0.40–1.28]	0.265	0.61 [0.27–1.40]	0.244
>65 years old	Reference		Reference	
**Coverage of health insurance**				
Yes	Reference		Reference	
No	2.18 [1.36–3.48]	0.001^*^	2.08 [1.12–3.87]	0.021^*^
**Comorbidity status**				
No comorbid	3.47 [2.06–5.85]	< 0.001^*^	2.03 [1.05–3.90]	0.034^*^
1–3 Comorbidities	Reference		Reference	
>4 Comorbidities	0.35 [0.95–1.28]	0.111	1.12 [0.65–19.72]	0,933
**Blood glucose control status**				
Regularly	Reference		Reference	
Irregularly	31.64 [16.57–60.42]	< 0.001^*^	23.61 [11.46–48.65]	0.000^*^
**Having general medical check-up**				
Yes	Reference		Reference	
No	4.22 [2.38–7.46]	< 0.001^*^	2.52 [1.12–5.36]	0.016^*^
**Education level**				
No education	2.87 [1.08–7.63]	0.035^*^	-	-
Elementary school	1.44 [0.85–2.43]	0.174	-	-
Junior high school	1.22 [0.63–2.35]	0.556	-	-
Senior high school	0.98 [0.21–4.69]	0.982	-	-
University	Reference		-	-
**Marital status**				
Not currently married	1.58 [0.90–2.77]	0.108	-	-
Currently married	Reference		-	-
**Economic status**				
Quintile 1	1.51 [0.79–2.88]	0.209	-	-
Quintile 2	1.38 [0.71–2.70]	0.347	-	-
Quintile 3	1.61 [0.82–3.13]	0.163	-	-
Quintile 4	2.38 [1.10–5.13]	0.027	-	-
Quintile 5	Reference		-	-
**Residency**				
Rural	2.73 [1.57–4.76]	< 0.001^*^	-	-
Urban	Reference		-	-
**Depressive symptoms status**				
Depressed	1.49 [0.79–2.79]	0.216	-	-
Not depressed	Reference			

^a^Goodness-of-fit p-value of final model: 0.552; pseudo-R-squared: 34.71%.

^b^Final multivariate model.

^*^p < 0.05 at the 5% level of significance.

## 4. Discussion

This study revealed that more than three-quarters of DM patients had low awareness of their medication therapy. This implies that only one out of four patients had high awareness of their medication therapy. Age and healthcare insurance coverage are sociodemographic factors associated with awareness of DM medication, whereas blood glucose monitoring status, comorbidity status, and having a routine medical check-up are behavioural factors associated with awareness of DM medication.

We observed that the young (26–35 years old) and middle (36–45 years old) adulthood were associated with low awareness of DM medication, similar to what a Malaysian study revealed (34). This might be due to the old misconception that DM is a disease that primarily only affects the elderly (35). However, the prevalence of type 2 diabetes in adolescents and adults is dramatically increasing due to unhealthy lifestyles and obesity, which has a more aggressive disease profile, leading to premature complications that affect the quality of life and long-term outcomes (36).

In this study, coverage of health insurance and awareness of DM medication were observed to be in significant association. Individuals with no health insurance were twice as likely to have low awareness of their medication therapy when compared with those who have health insurance. This might be because self-paying DM patients may have high substantial medical expenses or financial issues. In turn, this may lead them to forego diabetic care as well as DM medication that would otherwise help them survive their conditions (37). Another possible explanation for this is that patients without medical insurance more likely skip regular medical care (38) and do not acquire better education, which may lead to low awareness of DM medication therapy.

We further observed that the number of comorbidities had a significant association with awareness of DM medication. This study revealed that individuals with no comorbidities were twice as likely to have low awareness of their DM medication when compared with those with 1–3 comorbidities. This finding is in line with the results of a study conducted in Malaysia reporting that patients with comorbidities had a high level of awareness; however, their level of self-care practise for diabetes remained low (39). Other recent qualitative research showed that patients with no comorbidities felt they may have prevented disease progression had they been given a more detailed explanation of their situation earlier as they were unaware of the risk factors, complications, and comorbidities (40).

DM individuals with irregular blood glucose monitoring likely had low awareness of their medication therapy when compared with individuals with regular blood glucose monitoring. The possible reason is perhaps that patients with regular blood glucose monitoring were more conscious of the consequences of not taking the drugs appropriately (41). DM patients who never had general medical check-ups were twice as likely to have low awareness of DM medication when compared with those who had had a general medical check-up. This might be because general medical check-ups provide health-related information, help identify issues early, assist in planning treatments as well as improve the awareness of medication (42).

A majority of the sociodemographic factors were not associated with low awareness of DM medication. Sociodemographic factors, such as marital status, may be overly generic when predicting an individual's DM medication awareness. A study revealed no correlation between gender and low awareness of DM medication (43). However, males were reported to be less aware of DM than females were (44, 45). Furthermore, in our study, educational level is not related to medication awareness as health literacy may be more essential than educational level (46). By contrast, another study found individuals with higher educational levels to have more awareness (47). The current study also found residency not to be associated with low awareness of DM medication, contradicting the results of a previous study, which reported a correlation between urban residence and high awareness of DM medication as urban residents seek therapy more often and have easier access to care (48). Moreover, religiosity was not associated with DM medication awareness in this study. This finding is contrary to the results of previous studies suggesting that, in terms of providing assistance and coping with a disease condition, religiosity played a significant role (49, 50). We further observed that depressive symptoms, happiness, insomnia and satisfaction with healthcare are not associated with low awareness of DM medication therapy. Previous studies have reported that depressive symptoms (51), insomnia (52), and happiness (53) were not associated with awareness of DM medication, which is in line with the present results. By contrast, in previous studies, the individuals more satisfied with their healthcare were possibly more aware of their DM medication (54, 55).

**Figure 1:**
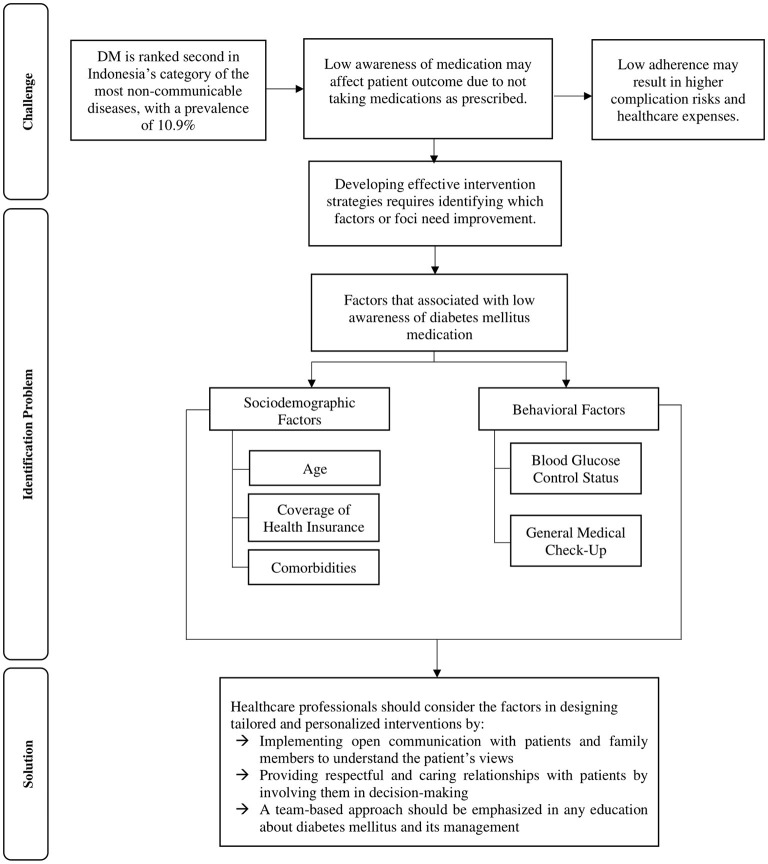
Problems, challenges and solutions to increase the level of awareness of diabetes mellitus medication in Indonesia.

The awareness of DM patients about their medication is crucial for ensuring that they take their DM medication as prescribed to avoid any complications or associated morbidities and mortality (56). These findings may help us understand the significance of the issue of awareness of DM medication as well as offer potential solutions. During the early stages of the disease, it may be necessary for patients to be better informed to increase their awareness of DM medication, particularly among those at high risk of developing comorbidities and complications (57). To raise patients' awareness of DM medication, healthcare professionals are essential information resources and play a leading role in the awareness-raising effort (58). Patient education can be improved by first determining the individual's learning needs and then providing them with individualised educational interventions tailored to meet their requirements (59). This study, thus, advocates for better management of DM by inquiring about medication adherence during clinical consultations and improving the quality of DM care. Furthermore, patient education, counselling and behavioural support are critical for achieving successful DM medication therapy. These tailored interventions could be effective in clarifying misconceptions and help clear up any misunderstandings to increase their level of awareness (60). In addition, healthcare professionals should monitor blood glucose, perform general medical check-ups regularly, prioritise patient satisfaction by ensuring that they receive appropriate care and establish a respectful and caring relationship with patients by involving them in decision-making (61). The current findings may be useful as a point of reference for healthcare professionals, addressing factors related to low awareness of DM medication (Figure 1).

Until now, the present study appears to be the first to assess the awareness of DM medication and its associated factors in Indonesia. The study's strength is that we used the IFLS data, which represents 83% of the Indonesian population with an attrition rate of only 6%. The IFLS provides numerous benefits, including large samples, which are relatively heterogenous, less expensive than collecting new data and representative of the Indonesia setting. Besides, in this analysis, individuals were included on the basis of an objective measurement of HbA1c >6.5%, providing a more objective individual selection and avoiding selection bias. Despite its strengths, the study certainly has certain limitations related to methodological issues. First, notably, the cross-sectional design of the study precludes any causal inferences regarding the relationship between sociodemographic and behavioural factors and low awareness of DM medication. Second, since we performed a complete case analysis, it may have reduced the statistical power, probably increasing the possibility of bias in our estimation, which might be an overestimation or underestimation of conclusions. Third, we have a wide CI value, indicating a greater likelihood of uncertainty regarding whether we have precisely estimated the strength of the association. Fourth, this study was at risk to recall bias due to disparities in accuracy in recalling past events based on self-reported answers from several variable independents. Fifth, this study was unable to distinguish between DM types 1 and 2. Sixth, as we relied on a secondary database that provides binary outcomes for the awareness, it might not be adequate to explain the multidimensional aspects of behavioural science (62). Further studies are needed to consider these aspects when assessing awareness of medication. Seventh, our model's overall association was low, indicating the possibility of other unmeasured factors influencing the low awareness of DM medication, such as another comorbid disease such as kidney disease (63), healthy lifestyle (64), education about DM (65), the number of medicines in the therapy (66), duration of DM (67), ethnic background (68) or medication beliefs (69).

## 5. Conclusion

Healthcare professionals should monitor blood glucose, perform general medical check-ups regularly, prioritise patient satisfaction by ensuring that they receive appropriate care and establish a respectful and caring relationship with patients by involving them in the decision-making process. Patient education can be improved by first determining the individual's learning needs and then providing them with individualised educational interventions tailored to meet their requirements in order to improve their awareness of DM medication. Therefore, our findings reveal the need to develop intervention strategies targeting those who irregularly monitor their blood glucose level; who irregularly undergo general medical check-ups, with multiple comorbidities; who have no health insurance coverage and who are young.

## Data availability statement

Publicly available datasets were analysed in this study. This data can be found here: https://www.rand.org/well-being/social-and-behavioral-policy/data/FLS/IFLS.html.

## Ethics statement

The Ethical Review Boards of the RAND's Human Subjects Protection Committee (s0064-06-01-CR01) approved IFLS research. Research Ethics Committee of Universitas Padjadjaran, Indonesia, waived the requirement because this study uses anonymous data from the IFLS.

## Author contributions

QK wrote the first draft of this manuscript. SA and RA participated in the design of the study. QK and SA participated in data analysis and interpretation. QK, SA, and RA revised the manuscript. All authors approved the final manuscript.
